# Long-term exposure to road traffic noise and stroke incidence: a Danish Nurse Cohort study

**DOI:** 10.1186/s12940-021-00802-2

**Published:** 2021-11-06

**Authors:** Tom Cole-Hunter, Christian Dehlendorff, Heresh Amini, Amar Mehta, Youn-Hee Lim, Jeanette T. Jørgensen, Shuo Li, Rina So, Laust H. Mortensen, Rudi Westendorp, Barbara Hoffmann, Elvira V. Bräuner, Matthias Ketzel, Ole Hertel, Jørgen Brandt, Steen Solvang Jensen, Jesper H. Christensen, Camilla Geels, Lise M. Frohn, Claus Backalarz, Mette K. Simonsen, Steffen Loft, Zorana J. Andersen

**Affiliations:** 1 Environmental Epidemiology Group, Section of Environmental Health, Department of Public Health, Faculty of Health and Medical Sciences, University of Copenhagen, Copenhagen, Denmark; 2 Centre for Air Pollution, Energy, and Health Research, University of New South Wales, Sydney, NSW Australia; 3 Statistics and Data Analysis, Danish Cancer Society Research Center, Copenhagen, Denmark; 4 Denmark Statistics, Copenhagen, Denmark; 5 Section of Epidemiology, Department of Public Health, Faculty of Health and Medical Sciences, University of Copenhagen, Copenhagen, Denmark; 6 Center for Healthy Aging, University of Copenhagen, Copenhagen, Denmark; 7 Institute for Occupational, Social and Environmental Medicine, Centre for Health and Society, Medical Faculty, Heinrich-Heine-University of Düsseldorf, Düsseldorf, Germany; 8 Department of Growth and Reproduction, Rigshospitalet, University of Copenhagen, Copenhagen, Denmark; 9 Department of Environmental Science, Aarhus University, Roskilde, Denmark; 10 Global Centre for Clean Air Research (GCARE), University of Surrey, Guildford, UK; 11 Department of Bioscience, Aarhus University, Roskilde, Denmark; 12 DELTA Acoustics, Hørsholm, Denmark; 13 Diakonissestiftelsen, Frederiksberg, Denmark; 14 The Parker Institute, Copenhagen University Hospital, Bispebjerg and Frederiksberg, Denmark

**Keywords:** Road traffic noise, Air pollution, NO_2_, PM_2.5_, PM_10_, Stroke, Ischemic, Hemorrhagic, Cohort

## Abstract

**Background:**

Road traffic noise has been linked to increased risk of ischemic heart disease, yet evidence on stroke shows mixed results. We examine the association between long-term exposure to road traffic noise and incidence of stroke, overall and by subtype (ischemic or hemorrhagic), after adjustment for air pollution.

**Methods:**

Twenty-five thousand six hundred and sixty female nurses from the Danish Nurse Cohort recruited in 1993 or 1999 were followed for stroke-related first-ever hospital contact until December 31st, 2014. Full residential address histories since 1970 were obtained and annual means of road traffic noise (L_den_ [dB]) and air pollutants (particulate matter with diameter < 2.5 μm and < 10 μm [PM_2.5_ and PM_10_], nitrogen dioxide [NO_2_], nitrogen oxides [NOx]) were determined using validated models. Time-varying Cox regression models were used to estimate hazard ratios (HR) (95% confidence intervals [CI]) for the associations of one-, three-, and 23-year running means of L_den_ preceding stroke (all, ischemic or hemorrhagic), adjusting for stroke risk factors and air pollutants. The World Health Organization and the Danish government’s maximum exposure recommendations of 53 and 58 dB, respectively, were explored as potential L_den_ thresholds.

**Results:**

Of 25,660 nurses, 1237 developed their first stroke (1089 ischemic, 148 hemorrhagic) during 16 years mean follow-up. For associations between a 1-year mean of L_den_ and overall stroke incidence, the estimated HR (95% CI) in the fully adjusted model was 1.06 (0.98–1.14) per 10 dB, which attenuated to 1.01 (0.93–1.09) and 1.00 (0.91–1.09) in models further adjusted for PM_2.5_ or NO_2_, respectively. Associations for other exposure periods or separately for ischemic or hemorrhagic stroke were similar. There was no evidence of a threshold association between L_den_ and stroke.

**Conclusions:**

Long-term exposure to road traffic noise was suggestively positively associated with the risk of overall stroke, although not after adjusting for air pollution.

**Supplementary Information:**

The online version contains supplementary material available at 10.1186/s12940-021-00802-2.

## Background

More than 12 million new cases of stroke were reported worldwide in 2017, of which about half were fatal, accounting for approximately 11% of all global deaths [1]. Almost 1 million stroke deaths globally were attributed to environmental risk factors, with air pollution playing a major role [1, 2]. Road traffic noise has been suggested as a contributing environmental risk factor for stroke, but evidence remains mixed. Several studies from Denmark and the UK have suggested an association between long-term exposure to road traffic noise and stroke incidence [3–5]. Thacher and colleagues (2020) reported a statistically significant association between road traffic noise and stroke mortality in the Danish Diet, Cancer and Health cohort, which corroborates findings from two earlier studies on road traffic noise and stroke incidence in the same cohort by Sørensen and colleagues (2011, 2014). However, a larger number of studies, from the Netherlands, Sweden, and Germany show no association between road traffic noise and stroke incidence [6–10].

One reason for uncertainty in reported associations between road traffic noise and stroke incidence is possible confounding by exposure to traffic-related air pollution, which itself has been linked to stroke incidence and can be moderately correlated at street level with road traffic noise [11]. Indeed, findings from several previous analyses have suggested that the association between road traffic noise and stroke is confounded by traffic-related air pollutants including nitrogen dioxide (NO_2_) [5, 12] and nitrogen oxides (NO_x_) [4, 5, 11]. While air quality within the European Union is generally improving due to effective regulation, the European Environment Agency projects that traffic noise will continue to rise in Europe and is not yet regulated [13]. Therefore, more studies are required to strengthen the body of evidence associating traffic noise and stroke, recently rated in quality as low for the WHO Environmental Noise Guidelines for the European Region [14].

Stroke is a complex disease that has two major subtypes, ischemic (most prevalent: 80–90%) and hemorrhagic, which differ in etiology and risk-factors [12]. On the one hand, inflammation and oxidative stress, commonly associated with air pollution exposure, are the basis of most pathways for the progression of ischemic stroke. The promotion of atherosclerosis is one long-term pathway to ischemic stroke [15], but impaired glucose control [16, 17] and high blood pressure [10] are also promoting factors. Shorter term, increased blood coagulability and cardiac arrhythmias can lead to acute ischemic stroke events [18]. On the other hand, high blood pressure is the main pathophysiological pathway to hemorrhagic stroke [19]. All of these mechanisms overlap or can influence each other, and may be relevant for the outcomes of long-term noise exposure.

Only one previous study, by Cai and colleagues (2018) in the UK, analysed road traffic noise effects by these specific stroke sub-types, and found a stronger (although statistically non-significant) association with ischemic stroke (remaining robust with adjustment for air pollution, namely particulate matter [PM] and NO_2_) [9]. Air pollution studies in Denmark have suggested stronger (statistically significant) effects with ischemic rather than hemorrhagic stroke [4, 5, 20, 21], which is in line with the overall evidence on stroke and air pollution [22] as well as the established association between air pollution and ischemic cardiovascular diseases [18]. Here we examine the association between long-term exposures to road traffic noise for up to 23 years and incidence of stroke, overall and by sub-types (ischemic and hemorrhagic), while adjusting for fine and coarse PM (PM_2.5_ and PM_10_, respectively), NO_2_, and NO_x_.

## Methods

### Cohort description

The Danish Nurse Cohort (DNC) is a nationwide cohort initiated in 1993 by mailing a questionnaire to 23,170 female nurses who were > 44 years of age, of which 19,898 (86%) responded with a completed questionnaire to begin participation [23]. In 1999, the DNC recruited 8833 more nurses, after inviting both non-responding individuals from 1993 (*N* = 489) and new women aged > 45 years (*N* = 8344) [23]. The questionnaire included a detailed inquiry on individual lifestyle factors, body weight, hormone therapy, reproductive history, health perception, and psycho-social work environment, which have been described in detail previously [23].

Using the unique personal identification number, participants were linked to the Civil Registration System which provides a full residential address history and vital status from 1970 until 2014 (inclusive) [24].

### Stroke definition

Participants were linked to the Danish National Patient Registry (DNPR) to obtain information on hospital contact due to stroke, using either International Classification of Diseases (ICD) iteration 8 (ICD-8: 431, 432, 433, 434, 436) or ICD-10 (I61, I63, and I64) codes. Stroke incidence was defined as the first hospital contact (either inpatient, outpatient, or emergency room admission) with a stroke diagnosis after cohort baseline (in 1993 or 1999). Participants with a history of stroke at baseline were excluded. If the sub-type of stroke was coded as “unknown”, we assumed it to be an ischemic stroke due to its much higher prevalence compared to hemorrhagic stroke [1].

### Exposure assessment

#### Road traffic noise at residence

Road traffic noise levels at the residential address from 1970 onwards were modelled using the Nord2000 model, and expressed as the annual mean of a weighted 24 h average (L_den_), adding a 5 dB penalty to the evening (19:00–22:00 h) noise levels and a 10 dB penalty to the night-time (22:00–07:00 h) noise levels. The main input variables of this model are geocodes of the location, polygons of all surrounding buildings and the height of apartments above street level, traffic composition and speed, road lines with information on yearly average daily traffic, road type and properties (e.g. rural highway, motorway, road wider than 6 m, and other roads), and meteorology (air temperature, cloud cover, wind speed, and wind direction). Further details of the Nord2000 model have been published previously [20, 25].

#### Air pollution at residence

Residential PM_2.5_, PM_10_, NO_2_, and NO_x_ levels for the period 1979–2014 (PM since 1990) were modelled using the DEHM/UBM/AirGIS, a multi-scale and high-resolution (1 km × 1 km) Danish air pollution modeling system [26]. The system is comprised of three air pollution dispersion models, which include the Danish Eulerian Hemispheric Model (DEHM), used to assess the long-range transport components [27, 28], the Danish Urban Background Model (UBM), to estimate the local background on a 1 km × 1 km resolution grid overlaying Denmark [29], and the Operational Street Pollution Model (OSPM), which estimates the residential address’ front door concentrations [30, 31]. The details and performance evaluation of the models are available elsewhere [32, 33].

### Statistical analyses

In this analysis, we considered both residential 1-year and 3-year mean concentration levels for all of the above mentioned exposures, preceding stroke event. In addition, as the data for L_den_ and gaseous air pollutants were available for longer periods, we analyzed 23-year mean residential concentrations for NO_2_ and NO_x_ (i.e., 23-year mean residential concentrations for PM_2.5_ and PM_10_ were not available) preceding stroke event.

Cox proportional hazards regression model with age as underlying time scale was used to estimate the association between road traffic noise and the incidence of overall, ischemic, and hemorrhagic stroke, separately. Hazard ratios (HR) and 95% confidence intervals (CI) were reported per 10 dB (continuous) and per tertile (categorical) of noise exposure level.

We estimated associations between road traffic noise and stroke in six models with increasing level of adjustment. Our first model was the ‘crude’ model, adjusted only for age and calendar year of entry into the cohort (1993 or 1999). Our second model was additionally (‘fully’) adjusted for stroke risk factors at baseline, including leisure-time physical activity (low, medium, high), alcohol consumption (none [0 drinks/week], moderate [1–14 drinks/week], and heavy [≥15 drinks/week]), fruit consumption (daily, weekly, rarely), smoking (never, previous, or current smoker), and marital status (married, separated, divorced, never married, and widowed). Our third model was considered the main model, additionally adjusted for PM_2.5_ as it is the air pollutant most convincingly related to stroke. Variations of this third model were made by substituting PM_2.5_ with PM_10_, NO_2_ or NO_x_.

We evaluated the shapes of the exposure-response functions by: (1) visually inspecting plots of the restricted cubic splines, and; (2) performing a likelihood ratio test comparing the model with a restricted cubic spline versus a model assuming a linear relationship.

We then set-out a priori to explore potential L_den_ thresholds, according to the World Health Organization [34] and the Danish government [35] maximum exposure recommendations of 53 and 58 dB, respectively, by truncating the model input by these dB levels.

Finally, as performed previously [20], we explored effect modification for the association between L_den_ and ischemic stroke by factors that may heighten susceptibility (described in Table 1) including: levels of PM_2.5_ or NO_2_, level of physical activity, obesity, presence of hypertension, acute myocardial infarction, or diabetes mellitus, use of hormone replacement therapy, status of occupation and night-shift work, and degree of urbanicity.

**Table 1:** Participant characteristics at baseline (1993/1999) in total and by stroke sub-type

Parameter	Level	Total	By stroke sub-type			
			No stroke	All stroke	Ischemic	Hemorrhagic
**N (%)**		25,660 (100)	24,423 (95)	1237 (5)	1089 (4)	148 (1)
**Age,** baseline, mean (SD)		52.9 (7.9)	52.6 (7.7)	59.5 (9.2)	59.8 (9.3)	57.2 (8.5)
**BMI,** mean (SD)		23.7 (3.5)	23.7 (3.5)	23.8 (3.6)	23.8 (3.6)	23.9 (3.8)
**Leisure time physical activity**, n (%)	Low	1734 (6.8)	1619 (6.7)	115 (9.5)	107 (10.0)	8 (5.4)
	Medium	16,889 (66.5)	16,068 (66.5)	821 (67.5)	723 (67.6)	98 (66.7)
	High	6765 (26.6)	6485 (26.8)	280 (23.0)	239 (22.4)	41 (27.9)
**Fruit intake**, n (%)	Rarely	930 (3.7)	879 (3.7)	51 (4.2)	42 (3.9)	9 (6.3)
	Weekly	7244 (28.6)	6911 (28.7)	333 (27.6)	289 (27.1)	44 (30.8)
	Daily	17,112 (67.7)	16,288 (67.6)	824 (68.2)	734 (68.9)	90 (62.9)
**Alcoholic drink intake, weekly**, n (%)	None (0)	3907 (15.7)	3663 (15.4)	244 (20.4)	227 (21.7)	17 (11.6)
	Moderate (1–14)	15,303 (61.4)	14,608 (61.6)	695 (58.2)	598 (57.1)	97 (66.0)
	Heavy (≥15)	5713 (22.9)	5458 (23.0)	255 (21.4)	222 (21.2)	33 (22.4)
**Smoking status**, n (%)	Never	8574 (34.5)	8239 (34.8)	335 (28.6)	294 (28.5)	41 (29.1)
	Former	7474 (30.1)	7134 (30.2)	340 (29.0)	298 (28.9)	42 (29.8)
	Current	8783 (35.4)	8285 (35.0)	498 (42.5)	440 (42.6)	58 (41.1)
**Marital status**, n (%)	Married	17,815 (70.1)	17,096 (70.6)	719 (58.7)	632 (58.7)	87 (58.8)
	Separated	453 (1.8)	429 (1.8)	24 (2.0)	23 (2.1)	1 (0.7)
	Divorced	2987 (11.7)	2820 (11.7)	167 (13.6)	142 (13.2)	25 (16.9)
	Never married	2539 (10.0)	2378 (9.8)	161 (13.1)	139 (12.9)	22 (14.9)
	Widowed	1635 (6.4)	1481 (6.1)	154 (12.6)	141 (13.1)	13 (8.8)
**Hypertension**, n (%)	Yes	3253 (12.7)	2960 (12.1)	293 (23.8)	261 (24.1)	32 (21.6)
**AMI**, n (%)	Yes	179 (0.7)	150 (0.6)	29 (2.4)	28 (2.6)	1 (0.7)
**Diabetes mel.**, n (%)	Yes	311 (1.2)	279 (1.2)	32 (2.6)	30 (2.8)	2 (1.4)
**Shift work type**, n (%)	Day	12,488 (62.3)	12,086 (62.4)	402 (61.0)	343 (60.5)	59 (64.1)
	Evening	2028 (10.1)	1947 (10.0)	81 (12.3)	75 (13.2)	6 (6.5)
	Night	1124 (5.6)	1069 (5.5)	55 (8.3)	49 (8.6)	6 (6.5)
	Rotating	4401 (22.0)	4280 (22.1)	121 (18.4)	100 (17.6)	21 (22.8)
**Urbanicity degree**, n (%)	Urban	7798 (30.4)	7384 (30.2)	414 (33.5)	364 (33.4)	50 (33.8)
	Suburban	5881 (22.9)	5610 (23.0)	271 (21.9)	233 (21.4)	38 (25.7)
	Rural	11,976 (46.7)	11,424 (46.8)	552 (44.6)	492 (45.2)	60 (40.5)

*Abbreviations*: *AMI* Acute myocardial infarction, *BMI* Body mass index, *Diabetes mel.* Diabetes mellitus, *n* Absolute number, *SD* Standard deviation

**Fig. 1:**
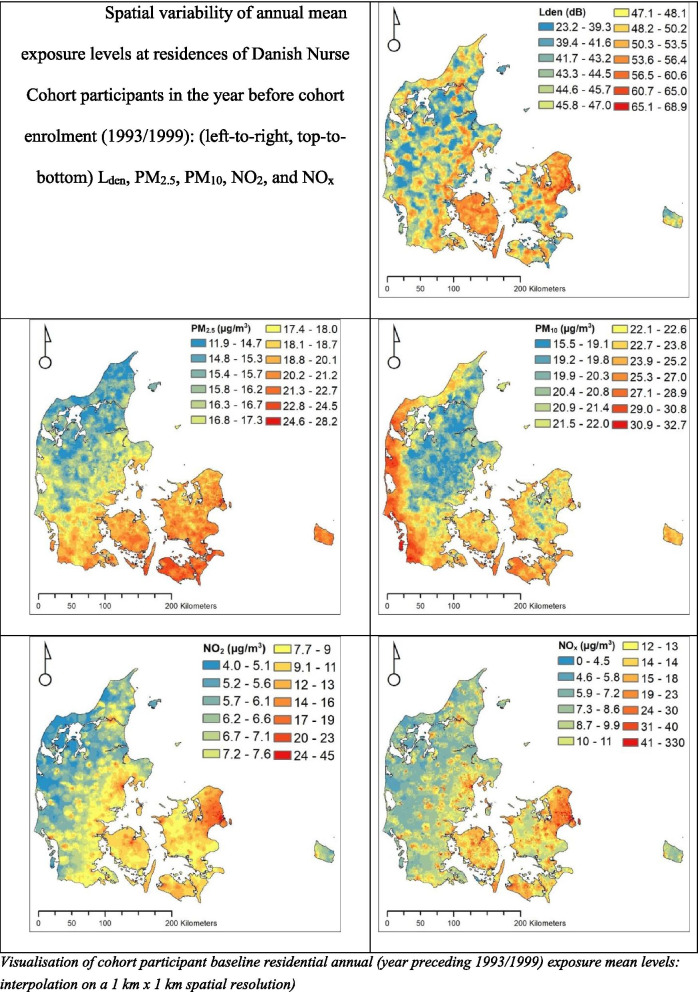
Spatial variability of annual mean exposure levels at residences of Danish Nurse Cohort participants in the year before cohort enrolment (1993/1999): (left-to-right, top-to-bottom) L_den_, PM_2.5_, PM_10_, NO_2_, and NO_x_

All statistical analyses were performed in R version 3.6.1 [36], using the following packages: knitr [37], tableone [38], rms [39], epi [40], and survival [41]. Exposure maps for each pollutant at cohort baseline, to illustrate spatial variations of exposures, were created using ArcGIS® software by ESRI (Fig. 1).

## Results

Out of a total 28,713 participants from both 1993 and 1999 cohorts, we excluded 151 who had stroke-related hospital contact prior to baseline and 2902 who did not have exposure or covariate information, leaving a total of 25,660 participants for final analysis. During a mean follow-up time of 16 years, 1237 (5%) participants developed stroke, resulting in an incidence rate of 248 per 100,000 person-years (Table 1). Mean age (SD) at cohort baseline was 52.9 (7.9) years (with stroke: 59.5 [9.2]; without stroke: 52.6 [7.7]) (Table 1).

Of the 1237 stroke cases, 1089 (88%) were ischemic and 148 (12%) were hemorrhagic. Compared to individuals without stroke, individuals with ischemic stroke were more likely to have hypertension, be current smokers, perform less physical activity, and be unmarried or widowed. Also compared to individuals without stroke, individuals with hemorrhagic stroke had similar prevalence of these risk factors except for performance of (similar) physical activity levels (Table 1).

Individuals with stroke were exposed to higher levels of L_den_ and all air pollutants than those without stroke (Table 2, Fig. 1). Individuals residing in areas with L_den_ above 58 dB showed a larger proportion of ischemic stroke compared to hemorrhagic or no stroke incidence (Table 2).

**Table 2:** Exposure characteristics at baseline (1993/1999) in total and by stroke sub-type

Parameter^**a**^	Level	Total	By stroke sub-type			
			No stroke	All stroke	Ischemic	Hemorrhagic
**N (%)**		25,660 (100)	24,423 (95.2)	1237 (4.8)	1089 (4.2)	148 (0.6)
**L**_**den**_ [dB]		52.72 (8.17)	52.68 (8.19)	53.52 (7.70)	53.58 (7.65)	53.10 (8.05)
**L**_**den**_**, categorical** [dB, tertiles], **n (%)**	< 48	5788 (22.6)	5539 (22.7)	249 (20.1)	217 (19.9)	32 (21.6)
	48–58	13,424 (52.3)	12,785 (52.3)	639 (51.7)	559 (51.3)	80 (54.1)
	> 58	6448 (25.1)	6099 (25.0)	349 (28.2)	313 (28.7)	36 (24.3)
**L**_**d**_ [dB]		50.48 (8.21)	50.44 (8.23)	51.33 (7.79)	51.39 (7.74)	50.88 (8.18)
**L**_**e**_ [dB]		48.12 (8.12)	48.08 (8.14)	48.94 (7.69)	49.00 (7.63)	48.48 (8.11)
**L**_**n**_ [dB]		44.59 (7.93)	44.55 (7.95)	45.32 (7.49)	45.39 (7.43)	44.83 (7.95)
**PM**_**2.5**_ [μg/m^3^]		19.77 (3.56)	19.70 (3.56)	21.22 (3.31)	21.27 (3.28)	20.88 (3.48)
**PM**_**10**_ [μg/m^3^]		23.64 (3.87)	23.56 (3.87)	25.20 (3.54)	25.21 (3.46)	25.10 (4.04)
**NO**_**2**_ [μg/m^3^]		12.65 (8.09)	12.57 (8.02)	14.09 (9.31)	14.05 (9.28)	14.37 (9.57)
**NO**_**x**_ [μg/m^3^]		19.20 (24.38)	19.01 (24.00)	23.09 (30.70)	22.84 (30.48)	24.95 (32.30)

*Abbreviations*: *dB* Decibel, *μg/m*^*3*^ Microgram per cubic meter, *n* Absolute number, *L*_*den*_ Annual mean day-evening-night (24-h) road traffic noise level, *L*_*d*_ Annual mean day-time (07:00–19:00 h) road traffic noise level, *L*_*e*_ Annual mean evening (19:00–23:00 h) road traffic noise level, *L*_*n*_ Annual mean night-time (23:00–07:00 h) road traffic noise level, *PM*_*2.5*_ Particulate matter with an aerodynamic diameter of < 2.5 μm, *PM*_*10*_ Particulate matter with an aerodynamic diameter of < 10 μm, *NO*_*2*_ Nitrogen dioxide, *NO*_*X*_ Nitrogen oxides

^a^Parameters are presented as mean (standard deviation), unless noted otherwise (i.e., N/n, %)

At baseline, the correlation of L_den_ with PM_2.5_ and PM_10_ was low (0.36, 0.24, respectively), while correlation with NO_2_ and NO_X_ was moderate (0.61, 0.50, respectively) (Table 3).

**Table 3:** Correlation (Spearman) matrix of exposure levels at baseline (1993/1999)

	L_**den**_	L_**d**_	L_**e**_	L_**n**_	PM_**2.5**_	PM_**10**_	NO_**2**_	NOx
**L**_**den**_	1							
**L**_**d**_	0.997	1						
**L**_**e**_	0.996	0.998	1					
**L**_**n**_	0.991	0.991	0.996	1				
**PM**_**2.5**_	0.360	0.365	0.363	0.357	1			
**PM**_**10**_	0.239	0.251	0.246	0.233	0.846	1		
**NO**_**2**_	0.606	0.619	0.616	0.601	0.649	0.510	1	
**NO**_**x**_	0.496	0.514	0.507	0.489	0.572	0.541	0.922	1

*Abbreviations*: *L*_*den*_ Annual mean day-evening-night (24-h) road traffic noise level, *L*_*d*_ Annual mean day-time (07:00–19:00 h) road traffic noise level, *L*_*e*_ Annual mean evening (19:00–23:00 h) road traffic noise level, *L*_*n*_ Annual mean night-time (23:00–07:00 h) road traffic noise level, *PM*_*2.5*_ Particulate matter with an aerodynamic diameter of < 2.5 μg/m^3^, *PM*_*10*_ Particulate matter with an aerodynamic diameter of < 10 μg/m^3^, *NO*_*2*_ Nitrogen dioxide, *NO*_*X*_ Nitrogen oxides

We found positive, but statistically non-significant associations for L_den_ exposure and incidence of overall stroke. The strongest (most suggestive) association in our crude model of overall stroke was found with one-year mean of L_den_ for which the HR (95% CI) per IQR increase of 10 dB was 1.06 (0.99, 1.13) (Table 4). The estimates were robust to adjustment for physical activity, smoking, marital status, and consumption of fruit and alcohol (fully-adjusted: 1.06 [0.98, 1.14]), but attenuated substantially after further adjustment for air pollution: PM_2.5_ (1.01 [0.93, 1.09]); PM_10_ (1.03 [0.95, 1.11]); NO_2_ (1.00 [0.91, 1.09]), and; NO_x_ (1.01 [0.92, 1.10]) (Table 4). Results were similar with 3-year and 23-year mean of L_den_ (Supplemental Tables S I and S II, respectively).

**Table 4:** Associations between 1-year mean road traffic noise level (continuous and categorical) and all incident stroke

Stroke sub-type	Noise variable	N	Model type					
			Crude	Fully-adj.	Fully-adj. + PM_**2.5**_	Fully-adj. + PM_**10**_	Fully-adj. + NO_**2**_	Fully-adj. + NO_**x**_
**All**	L_den_, continuous^a^	1237	1.06 (0.99–1.13)	1.06 (0.98–1.14)	1.01 (0.93–1.09)	1.03 (0.95–1.11)	1.00 (0.91–1.09)	1.01 (0.92–1.10)
	L_den_, cat: < 48 dB	249	[ref]	[ref]	[ref]	[ref]	[ref]	[ref]
	L_den_, cat: 48–58 dB	639	1.10 (0.95–1.27)	1.12 (0.96–1.31)	1.08 (0.92–1.28)	1.11 (0.94–1.30)	1.08 (0.91–1.27)	1.10 (0.93–1.29)
	L_den_, cat: > 58 dB	349	1.13 (0.96–1.32)	1.13 (0.95–1.34)	1.02 (0.85–1.23)	1.07 (0.89–1.28)	0.99 (0.80–1.22)	1.02 (0.84–1.24)
**Ischemic**	L_den_, continuous^a^	1089	1.06 (0.99–1.14)	1.06 (0.98–1.14)	1.00 (0.92–1.09)	1.03 (0.94–1.12)	0.98 (0.89–1.09)	1.00 (0.92–1.10)
	L_den_, cat: < 48 dB	217	[ref]	[ref]	[ref]	[ref]	[ref]	[ref]
	L_den_, cat: 48–58 dB	559	1.04 (0.89–1.22)	1.05 (0.89–1.24)	1.02 (0.86–1.21)	1.04 (0.88–1.24)	1.01 (0.85–1.21)	1.03 (0.87–1.23)
	L_den_, cat: > 58 dB	313	1.10 (0.93–1.31)	1.10 (0.92–1.32)	0.99 (0.81–1.21)	1.05 (0.86–1.27)	0.95 (0.76–1.19)	1.00 (0.81–1.23)
**Hemorrhagic**	L_den_, continuous^a^	148	1.04 (0.86–1.26)	1.06 (0.87–1.30)	1.05 (0.84–1.31)	1.04 (0.84–1.29)	1.10 (0.84–1.43)	1.03 (0.81–1.30)
	L_den_, cat: < 48 dB	32	[ref]	[ref]	[ref]	[ref]	[ref]	[ref]
	L_den_, cat: 48–58 dB	80	1.64 (1.04–2.57)	1.82 (1.10–3.02)	1.69 (1.02–2.82)	1.71 (1.03–2.84)	1.72 (1.03–2.87)	1.69 (1.01–2.80)
	L_den_, cat: > 58 dB	36	1.33 (0.81–2.18)	1.41 (0.81–2.45)	1.31 (0.74–2.34)	1.30 (0.74–2.30)	1.37 (0.73–2.58)	1.22 (0.67–2.23)

Model estimates are hazard ratios and 95% confidence intervals [HR (95% CI)]. Crude model: adjusted for age (calendar year / underlying time) and year of cohort entry (inclusion year: 1993/1999); Fully-adjusted model: Crude model + physical activity, marital status, alcohol, smoking, and fruit consumption

*Abbreviations*: *cat* Categorical (tertiles), *dB* Decibel, *Fully-adj.* Fully-adjusted, *L*_*den*_ Annual mean 24-h road traffic noise levels, *ref* Categorical reference

^a^10 dB increments of L_den_

**Fig. 2:**
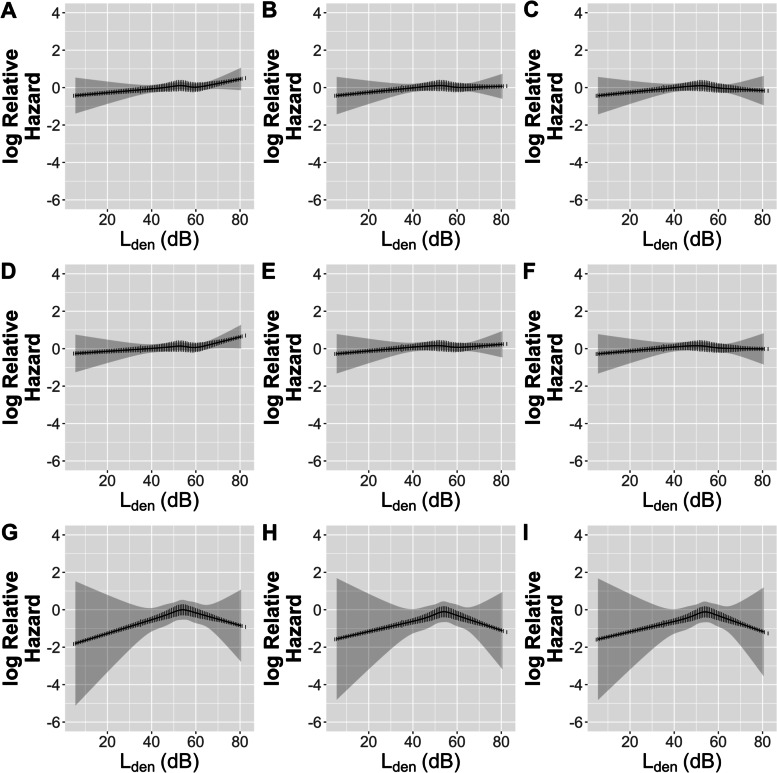
Association (restricted cubic spline) between 1-year mean L_den_ exposures and incident Stroke (all, ischemic, or hemorrhagic) among the Danish Nurse Cohort

We observed a mild non-monotonic exposure-response relationship for total and ischemic stroke, and an indication of a threshold above which the effect was not observed or mildly reversed for L_den_ (approximately 55 dB) for both 1-year and 3-year means: however, the reverse was observed as strong for hemorrhagic stroke (Fig. 2 and S I, respectively). The likelihood ratio test of non-linearity showed no statistically significant violation of linearity for any exposure variable or window except for the NO_x_ 3-year mean (*p* = 0.02); an anomaly, considering the 1-year (*p* = 0.21) and 23-year (*p* = 0.22) means (Fig. S II, Table S III). Truncation of L_den_ values above the health-indicated thresholds of 53 and 58 dB resulted in higher HRs for the crude and fully-adjusted models, however not when additionally adjusting for any air pollutant (Tables S IV and S V). There was no apparent effect modification of association between road traffic noise and stroke by any air pollutant, personal characteristic or coinciding environmental characteristic (Table S VI).

## Discussion

In this cohort study of Danish female nurses, we find weak support for associations between long-term exposure to road traffic noise and incidence of overall, ischemic or hemorrhagic stroke. To our knowledge, this is one of only a few studies that have evaluated this association separately for both sub-types of ischemic and hemorrhagic stroke, and adjusted for air pollution [42, 43]. While we observed suggestions of a positive association between continuous L_den_ and total or ischemic stroke, these were generally attenuated after adjustment for either of the air pollutants. Except for NO_2_/NO_x_, these air pollutants (PM_2.5_, PM_10_) were weakly positively correlated with L_den_. As such, the suggestion of an adverse effect of long-term road traffic noise on stroke outcome seems to be at least partly attributable to coinciding air pollution exposure. Testing for effect modification of the association between noise and incidence of ischemic stroke showed insignificant interaction between noise and air pollution.

Our finding that ischemic (rather than hemorrhagic) stroke was more suggestively associated with road traffic noise, based upon direction of hazard ratios and 95% confidence intervals from continuous exposure models, supports findings from the one previous noise analysis also using both specific stroke subtypes [9]; however, that previous analysis’ observation of an association was robust to adjustment for PM and NO_2_, while ours was not. Similarly, two studies found a significant positive association between road traffic noise and specifically ischemic stroke incidence [5, 44]. Héritier and colleagues also found significant positive associations between both road and air traffic noise levels with ischemic but not hemorrhagic stroke [43]. On the contrary, Seidler and colleagues investigated short-term exposure (daily average and maximum noise levels) of different transport sources to find a greater risk for hemorrhagic rather than ischemic stroke with railway and aircraft but not road traffic noise, postulating a link to hypertension from night-time noise events [42]. Other studies, however, do not specify risk according to stroke subtypes, which may explain the mixed findings for association with road traffic noise largely seen in the literature [3, 6–8]. Our finding that hemorrhagic stroke is significantly associated with moderate but not high levels of noise, based upon categorical exposure models, may be explained by the larger sample size for the former (*N* = 80) compared to the latter (*N* = 36) category for this diagnosis.

Our finding that the association between road traffic noise and overall stroke incidence is nullified when considering air pollution agrees with findings reported previously [6, 7, 43], most notably in a meta-analysis giving a pooled null effect [45]. Some studies, however, have still found a significant positive association between road traffic noise and overall stroke incidence after considering air pollution [4, 5, 46]. See supplemental Table S VII for a full overview of studies on associations between long-term exposure to road traffic noise and stroke.

Disagreement between studies of road traffic noise exposure and stroke incidence may be explained by a high correlation between exposures to emissions from common sources such as motorized traffic [11], previously seen as confounding associations to cardio- and cerebrovascular outcomes [5, 47]. In the current study, we observe moderate correlation between road traffic noise and the gaseous traffic-related air pollutants of NO_2_/NO_x_, but not particulate matter (PM_2.5_/PM_10_). When further adjusting noise models for air pollution, a stronger attenuation of the suggestion for association with stroke was observed for NO_2_/NO_x_ compared to PM_2.5_/PM_10_. Previous studies have shown that road traffic noise may confound the association between stroke incidence and any of these air pollutants: NO_2_ [5, 12], NO_x_ [4, 5, 11] and PM_2.5_ [9]. In general, the literature is much more exhaustive for the analysis of association between air pollution and stroke incidence, which challenges the interpretation of our findings.

Danish studies have found no association between overall stroke incidence and NO_x_ with or without adjustment for road traffic noise [4], or an association between ischemic stroke and road traffic noise independently of NO_2_; however, fatal stroke was associated with NO_2_ but not road traffic noise [5]. A more recent analysis, of the same cohort as our current study, observed that road traffic noise did not confound the positive association between overall stroke and PM_2.5_ [20]. Similarly, a German study found an association between stroke and PM_2.5_, which was not confounded by road traffic noise [48]. This independent effect of long-term PM_2.5_ exposure on the incidence of stroke has been highlighted in a recent meta-analysis (of 13 cohorts) [22] and other large cohort studies [49, 50]. Our suggestion of an association between all or ischemic stroke and road traffic noise was observed to be attenuated (confounded) similarly by either pollutant.

Regarding exposure window periods, we observe the suggestion for a larger effect of road traffic noise on stroke incidence when using the shortest (1-year) compared to longer (3-, 23-year) windows. However, when adjusting for air pollution (confounding), this temporal effect disappears. Moreover, we observe less confounding of air pollution on the association between stroke and noise in the 23-year window. Few studies have evaluated the sensitivity of road traffic noise and stroke association to the temporal scale of exposure assessment, and none have evaluated exposure windows as long as 23 years. The closest to achieving this evaluation are two studies also performed in Denmark, which both looked at 1-, 5- and 10-year exposure windows [5, 46]. Their results on this were opposing: Sørensen and colleagues found similar temporal effects to our present study (largest effect for 1-year) while Thacher and colleagues found the largest effect for 10-year windows.

Shorter term, experimental (laboratory) studies among humans have shown how increased blood pressure, heart rate variability and stress hormone release may initiate the pathway from noise exposure to ischemic heart disease and stroke [51]. The mechanism by which PM_2.5_ may attenuate the effect of road traffic noise on stroke, as we observed in the current study, has been alluded to in previous studies of cerebrovascular diseases. The Multi-Ethnic Study of Atherosclerosis and Air Pollution (MESA Air) has found long-term exposure to ambient PM_2.5_ to promotes atherosclerosis by significantly decreasing endothelial function [52] and increasing intima-medial thickness [53]. Other study findings suggest that exposure to ambient particles increases formation of peripheral thrombosis and atherosclerotic lesions through multiple pathways [54]. The mechanism by which NO_2_ or other ambient gases may modify the effect of road traffic noise on stroke is less known. Toxicological evidence suggests that NO_2_ reacts with airway surface fluid constituents to produce highly reactive protein and lipid oxidation products, subsequently causing inflammation through secondary reactions from damaged epithelial cells [55].

The strength of our study is that it is based on a nationwide cohort of nurses, with large contrasts in exposure to noise, information on stroke subtypes, as well as with detailed information on individual covariates. Since we only included middle-age female nurses, we have reduced confounding by socioeconomic status (expected to differ across age as a proxy for career stage). Taking advantage of the extensive Danish registers, we could define stroke incidence by sub-types and across an individual’s entire residential address history since 1970.

The limitations of our findings include a reduced generalizability (external validity) to younger individuals (< 44 years of age) and males, as well as populations in cities of higher air pollution or traffic-related exposure levels, which are not represented in our study. Previous studies on noise have generally shown differential effects in relation to age, sex and socioeconomic status, which challenges the external validity of our findings. Further, in general, there are known limitations of sound pressure level (L_den_) as a metric for noise exposure, such as how sound events are received to trigger a stress response or not (i.e., annoyance), and potentially depending on time of day (e.g., L_n_ exposure may be a more impactful risk factor for cardiovascular health due to sleep disturbance). Alternative metrics including intermittency ratio (measuring events) and other psychoacoustic measures, if available, add value to L_den_ in terms of peaks in noise above background levels and individual noise sensitivities [43, 56]. Finally, we lacked data on living (indoor) conditions at the residence, including bedroom orientation and window insulation. All of these factors, which we could not account for, could have contributed to exposure misclassification and the weak effects we observed.

## Conclusions

In conclusion, in a nationwide cohort of Danish nurses aged 44 years and older, we found a suggestive positive association between road traffic noise and total or specifically ischemic (but not hemorrhagic) stroke, which was attenuated when adjusting for air pollution. This attenuation could be explained by potential exposure misclassification, leading to inconclusive results, however is not necessarily proof of absence of effect of noise on stroke.

## Supplementary Information

**Additional file 1: Figure S I.** Association (restricted cubic spline) between 3-year mean L_den_ exposures and incident Stroke (all, ischemic, or hemorrhagic) among the Danish Nurse Cohort. **Figure S II.** Restricted cubic spline plots to check linearity in adjusted models. **Table S I.** Associations between 3-year mean road traffic noise level (continuous and categorical) and incident Stroke (all, ischemic, hemorrhagic) among the Danish Nurse Cohort. **Table S II.** Associations between 23-year mean road traffic noise level (continuous and categorical) and incident Stroke (all, ischemic, hemorrhagic) among the Danish Nurse Cohort. **Table S III.** Likelihood ratio tests of significance for non-linear exposures. **Table S IV.** Associations between 1-, 3-, and 23-year mean road traffic noise level (53 dB cut-off) and incident Stroke (all, ischemic, hemorrhagic) among the Danish Nurse Cohort. **Table S V.** Associations between 1-, 3-, and 23-year mean road traffic noise level (58 dB cut-off) and incident Stroke (all, ischemic, hemorrhagic) among the Danish Nurse Cohort. **Table S VI.** Effect modification of the association between L_den_ (continuous, 1-year mean, per 10 dB increase) and incidence of ischemic stroke in the Danish Nurse Cohort. **Table S VII.** Overview of studies on associations between long-term exposure to road traffic noise and stroke.

## Data Availability

The dataset supporting the conclusions of this article will be archived in the Danish Data Archive (https://www.sa.dk/en/about-us/danish-national-archives), from which data can be accessed following the rules of the Danish legislation.

## Abbreviations

dB: Decibel
DNC: Danish Nurse Cohort
DNPR: Danish National Patient Registry
HR: Hazard ratio
ICD: International Classification of Diseases
L_den_: Annual mean day-evening-night (24-h) road traffic noise level
L_d_: Annual mean day-time (07:00–19:00 h) road traffic noise level
L_e_: Annual mean evening (19:00–23:00 h) road traffic noise level
L_n_: Annual mean night-time (23:00–07:00 h) road traffic noise level
PM_2.5_: Particulate matter with an aerodynamic diameter of < 2.5 μm
PM_10_: Particulate matter with an aerodynamic diameter of < 10 μm
NO_2_: Nitrogen dioxide
NO_X_: Nitrogen oxides
SD: Standard deviation
μg/m^3^: Microgram per cubic meter
