# Supplementary material for: Long-term exposure to road traffic noise and stroke incidence: a Danish Nurse Cohort study
Source: Environ Health. 2021 Nov 6;20:115. doi: 10.1186/s12940-021-00802-2 (PMC8571835; doi:10.1186/s12940-021-00802-2)
Supplement: Supplementary file 1 — Additional file 1: Figure S I. Association (restricted cubic spline) between 3-year mean Lden exposures and incident Stroke (all, ischemic, or hemorrhagic) among the Danish Nurse Cohort. Figure S II. Restricted cubic spline plots to check linearity in adjusted models. Table S I. Associations between 3-year mean road traffic noise level (continuous and categorical) and incident Stroke (all, ischemic, hemorrhagic) among the Danish Nurse Cohort. Table S II. Associations between 23-year mean road traffic noise level (continuous and categorical) and incident Stroke (all, ischemic, hemorrhagic) among the Danish Nurse Cohort. Table S III. Likelihood ratio tests of significance for non-linear exposures. Table S IV. Associations between 1-, 3-, and 23-year mean road traffic noise level (53 dB cut-off) and incident Stroke (all, ischemic, hemorrhagic) among the Danish Nurse Cohort. Table S V. Associations between 1-, 3-, and 23-year mean road traffic noise level (58 dB cut-off) and incident Stroke (all, ischemic, hemorrhagic) among the Danish Nurse Cohort. Table S VI. Effect modification of the association between Lden (continuous, 1-year mean, per 10 dB increase) and incidence of ischemic stroke in the Danish Nurse Cohort. Table S VII. Overview of studies on associations between long-term exposure to road traffic noise and stroke. [file 12940_2021_802_MOESM1_ESM.docx]

**SUPPLEMENTAL**

**Long-term exposure to road traffic noise and stroke incidence: A Danish Nurse Cohort study**

Tom Cole-Hunter^1,2,*^, Christian Dehlendorff^3^, Heresh Amini^1^, Amar Mehta^4,5^, Youn-Hee Lim^1^, Jeanette T. Jørgensen^1^, Shuo Li^1^, Rina So^1^, Laust H Mortensen^5,6^, Rudi Westendorp^5,6^, Barbara Hoffmann^7^, Elvira V. Bräuner^8^, Matthias Ketzel^9,10^, Ole Hertel^11^, Jørgen Brandt^9^, Steen Solvang Jensen^9^, Jesper H. Christensen^9^, Camilla Geels^9^, Lise M. Frohn^9^, Claus Backalarz^12^, Mette K. Simonsen^13,14^, Steffen Loft^1^, Zorana J. Andersen^1^

^1^ Environmental Epidemiology Group, Section of Environmental Health, Department of Public Health, Faculty of Health and Medical Sciences, University of Copenhagen, Copenhagen, Denmark

^2^ Centre for Air pollution, Energy, and health Research, University of New South Wales, Sydney, NSW, Australia

^3^ Statistics and Data analysis, Danish Cancer Society Research Center, Copenhagen, Denmark

^4^ Denmark Statistics, Copenhagen, Denmark

^5^ Section of Epidemiology, Department of Public Health, Faculty of Health and Medical Sciences, University of Copenhagen, Copenhagen, Denmark

^6^ Center for Healthy Aging, University of Copenhagen, Copenhagen, Denmark

^7^ Institute for Occupational, Social and Environmental Medicine; Centre for Health and Society, Medical Faculty, Heinrich-Heine-University of Düsseldorf, Düsseldorf, Germany

^8^ Department of Growth and Reproduction, Rigshospitalet, University of Copenhagen, Denmark

^9^ Department of Environmental Science, Aarhus University, Roskilde, Denmark

^10^ Global Centre for Clean Air Research (GCARE), University of Surrey, United Kingdom

^11^ Department of Bioscience, Aarhus University, Roskilde, Denmark

^12^ DELTA Acoustics, Hørsholm, Denmark

^13^ Diakonissestiftelsen, Frederiksberg, Denmark

^14^ The Parker Institute, Copenhagen University Hospital, Bispebjerg and Frederiksberg, Denmark

^*^ Corresponding author: Tom Cole-Hunter, Environmental Epidemiology Group, Section of Environmental Health, Department of Public Health, Faculty of Health and Medical Sciences, University of Copenhagen, Copenhagen, Denmark. Tel: +45 35 32 76 69, Email: [t.cole-hunter@sund.ku.dk](mailto:t.cole-hunter@sund.ku.dk)

**Figure S I ­**– Association (restricted cubic spline) between 3-year mean L_den_ exposures and incident Stroke (all, ischemic, or hemorrhagic) among the Danish Nurse Cohort

**
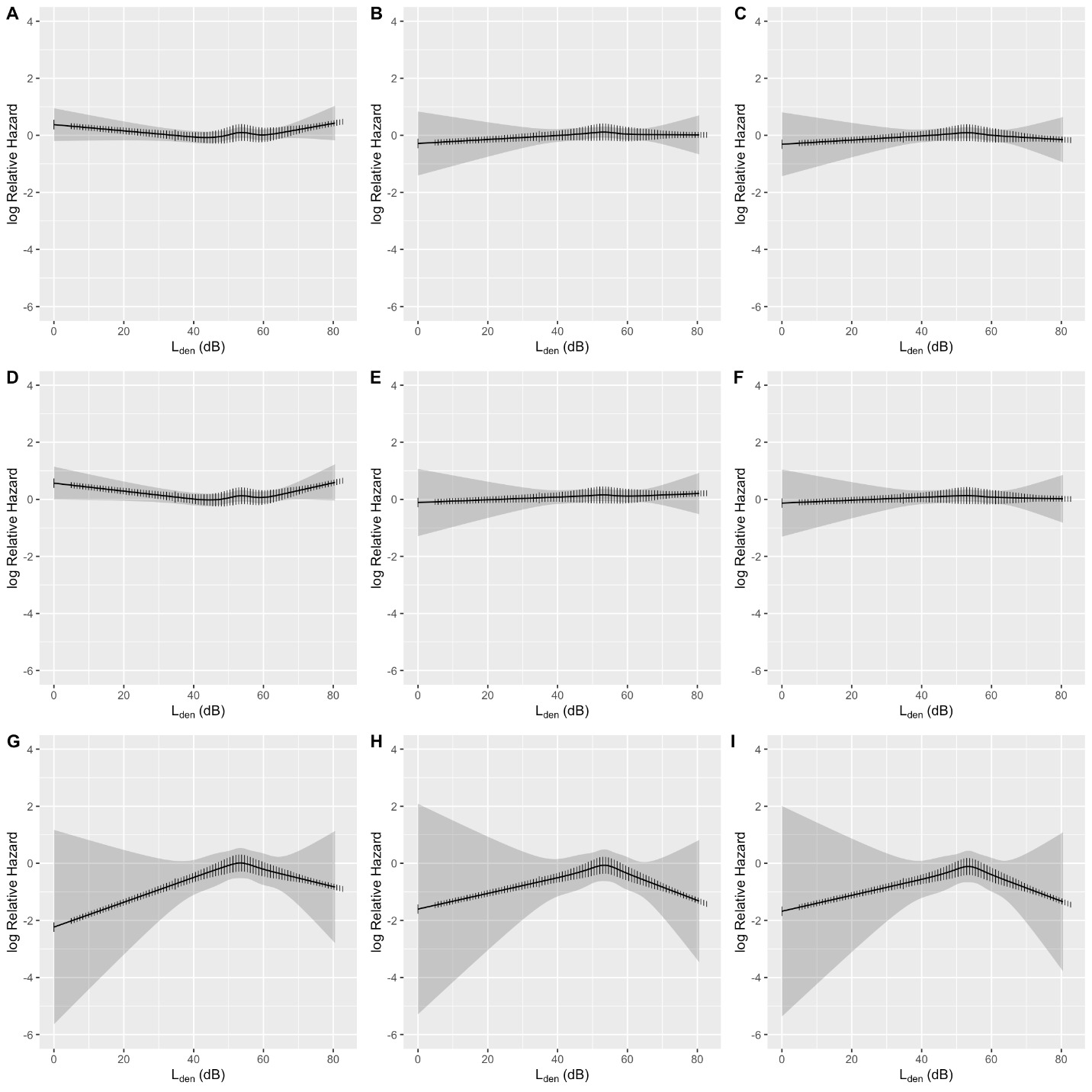
**

*A: All stroke; B: All stroke, fully adjusted for PM_2.5_; C: All stroke, fully adjusted for NO_2_; D-F: Ischemic stroke, adjusted as A-C; G-I: Hemorrhagic stroke, adjusted as A-C.*

**Figure S II –** Restricted cubic spline plots to check linearity in adjusted models


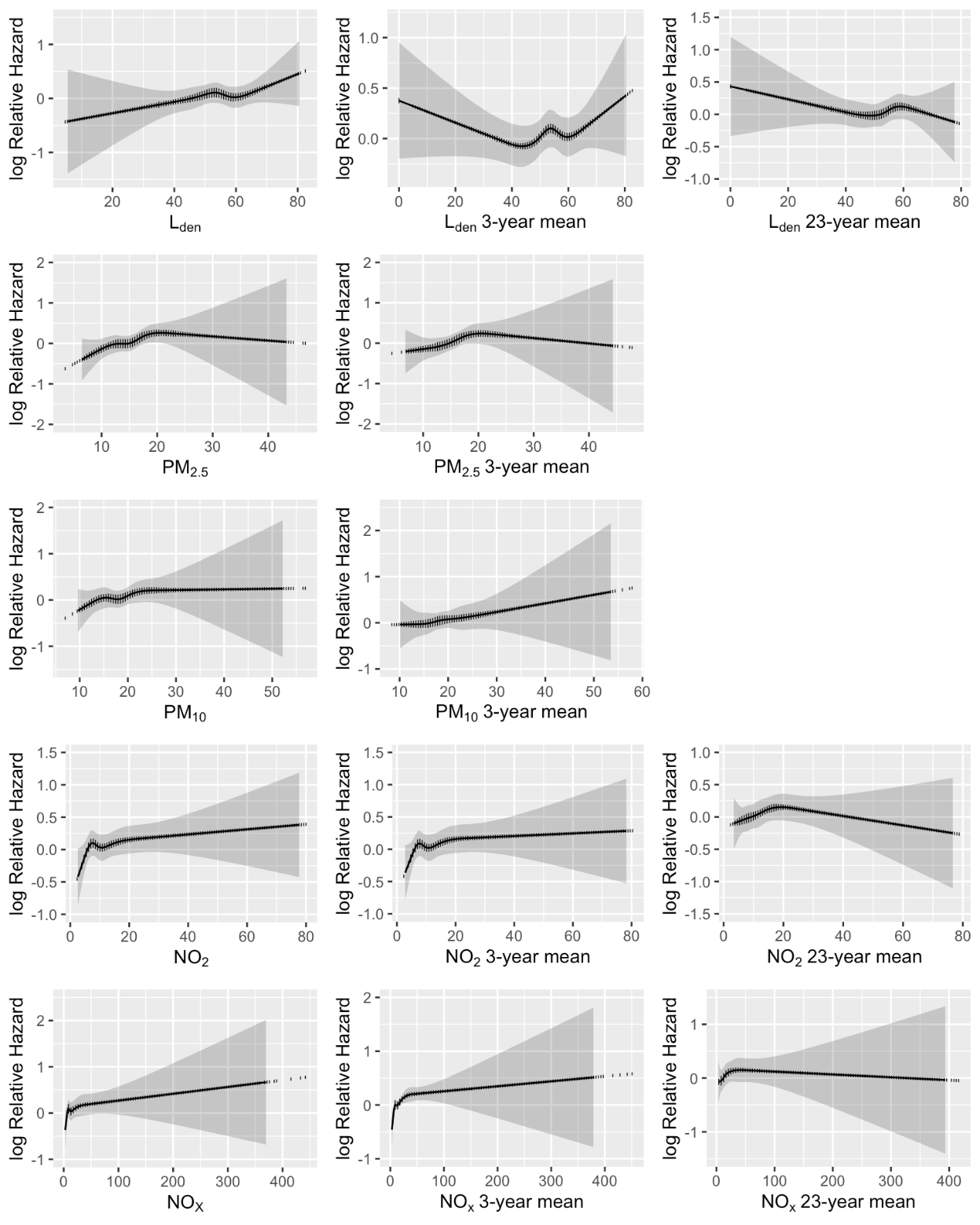


*We evaluated the shapes of exposure-response functions by plotting restricted cubic. If a plateau was obvious from the plot of the restricted cubic spline, the exposure values were truncated corresponding to a piecewise linear relationship with two segments, the first having a slope and the other forced to be flat, and the association was re-investigated.*

**Table S I –** Associations between 3-year mean road traffic noise level (continuous and categorical) and incident Stroke (all, ischemic, hemorrhagic) among the Danish Nurse Cohort

| **Stroke sub-type** | **Noise variable** | **N** | **Model type** | | | | | |
| --- | --- | --- | --- | --- | --- | --- | --- | --- |
|  |  |  | **Crude** | **Fully-adjusted** | **Fully-adjusted + PM_2.5_** | **Fully-adjusted + PM_10_** | **Fully-adjusted + NO_2_** | **Fully-adjusted + NO_x_** |
| **All** | L_den_, continuous^*^ | 1237 | 1.02 (0.96-1.09) | 1.02 (0.95-1.09) | 1.01 (0.93-1.10) | 1.04 (0.96-1.12) | 1.01 (0.92-1.11) | 1.01 (0.93-1.11) |
|  | L_den_, cat: <48 dB | 249 | [ref] | [ref] | [ref] | [ref] | [ref] | [ref] |
|  | L_den_, cat: 48-58 dB | 639 | 1.04 (0.90-1.20) | 1.05 (0.90-1.22) | 1.03 (0.88-1.22) | 1.06 (0.90-1.24) | 1.03 (0.88-1.21) | 1.05 (0.89-1.23) |
|  | L_den_, cat: >58 dB | 349 | 1.07 (0.92-1.25) | 1.06 (0.90-1.25) | 0.98 (0.81-1.18) | 1.03 (0.86-1.24) | 0.95 (0.78-1.18) | 0.98 (0.81-1.19) |
| **Ischemic** | L_den_, continuous^*^ | 1089 | 1.02 (0.96-1.09) | 1.01 (0.95-1.09) | 1.02 (0.93-1.11) | 1.04 (0.96-1.14) | 1.00 (0.91-1.11) | 1.02 (0.93-1.12) |
|  | L_den_, cat: <48 dB | 217 | [ref] | [ref] | [ref] | [ref] | [ref] | [ref] |
|  | L_den_, cat: 48-58 dB | 559 | 0.98 (0.85-1.14) | 0.98 (0.84-1.16) | 0.98 (0.83-1.17) | 1.01 (0.85-1.19) | 0.98 (0.82-1.16) | 0.99 (0.84-1.18) |
|  | L_den_, cat: >58 dB | 313 | 1.06 (0.90-1.24) | 1.05 (0.88-1.25) | 0.97 (0.80-1.19) | 1.03 (0.85-1.25) | 0.94 (0.75-1.17) | 0.98 (0.80-1.20) |
| **Hemorrhagic** | L_den_, continuous^*^ | 148 | 1.05 (0.88-1.26) | 1.06 (0.88-1.28) | 0.98 (0.79-1.23) | 0.99 (0.80-1.23) | 1.05 (0.81-1.36) | 0.99 (0.78-1.26) |
|  | L_den_, cat: <48 dB | 32 | [ref] | [ref] | [ref] | [ref] | [ref] | [ref] |
|  | L_den_, cat: 48-58 dB | 80 | 1.63 (1.04-2.53) | 1.69 (1.04-2.73) | 1.49 (0.92-2.43) | 1.54 (0.95-2.51) | 1.53 (0.93-2.50) | 1.52 (0.93-2.47) |
|  | L_den_, cat: >58 dB | 36 | 1.23 (0.75-2.02) | 1.22 (0.71-2.09) | 1.02 (0.57-1.83) | 1.06 (0.60-1.85) | 1.09 (0.58-2.04) | 1.01 (0.56-1.82) |

*Abbreviations: cat, categorical (tertiles); dB, decibel; L_den_, annual mean 24-hour road traffic noise levels; N, sample size; ref, categorical reference in model
Model estimates are hazard ratios and 95% confidence intervals [HR (95% CI)]
^*^ 10 dB increments of L_den_Crude model: adjusted for age (calendar year / underlying time) and year of cohort entry (inclusion year: 1993/1999);
Fully-adjusted model: Crude model + physical activity, marital status, alcohol, smoking, and fruit consumption.*

**Table S II –** Associations between 23-year mean road traffic noise level (continuous and categorical) and incident Stroke (all, ischemic, hemorrhagic) among the Danish Nurse Cohort

| **Stroke sub-type** | **Noise variable** | **N** | **Model type** | | | | | |
| --- | --- | --- | --- | --- | --- | --- | --- | --- |
|  |  |  | **Crude** | **Fully-adjusted** | **Fully-adjusted + PM_2.5_** | **Fully-adjusted + PM_10_** | **Fully-adjusted + NO_2_** | **Fully-adjusted + NO_x_** |
| **All** | L_den_, continuous^*^ | 1237 | 1.04 (0.97-1.11) | 1.02 (0.95-1.10) | [unavail.] | [unavail.] | 1.01 (0.91-1.12) | 1.02 (0.93-1.12) |
|  | L_den_, cat: <48 dB | 249 | [ref] | [ref] | [ref] | [ref] | [ref] | [ref] |
|  | L_den_, cat: 48-58 dB | 639 | 1.02 (0.89-1.16) | 0.97 (0.84-1.13) | [unavail.] | [unavail.] | 0.95 (0.81-1.11) | 0.96 (0.82-1.12) |
|  | L_den_, cat: >58 dB | 349 | 1.11 (0.95-1.29) | 1.05 (0.89-1.25) | [unavail.] | [unavail.] | 0.96 (0.78-1.19) | 0.99 (0.81-1.20) |
| **Ischemic** | L_den_, continuous^*^ | 1089 | 1.05 (0.97-1.13) | 1.03 (0.95-1.12) | [unavail.] | [unavail.] | 1.04 (0.93-1.16) | 1.05 (0.95-1.17) |
|  | L_den_, cat: <48 dB | 217 | [ref] | [ref] | [ref] | [ref] | [ref] | [ref] |
|  | L_den_, cat: 48-58 dB | 559 | 1.04 (0.90-1.21) | 1.01 (0.86-1.18) | [unavail.] | [unavail.] | 0.99 (0.84-1.17) | 1.00 (0.85-1.19) |
|  | L_den_, cat: >58 dB | 313 | 1.15 (0.97-1.36) | 1.10 (0.92-1.32) | [unavail.] | [unavail.] | 1.01 (0.80-1.27) | 1.04 (0.85-1.29) |
| **Hemorrhagic** | L_den_, continuous^*^ | 148 | 0.95 (0.78-1.15) | 0.93 (0.76-1.14) | [unavail.] | [unavail.] | 0.85 (0.65-1.12) | 0.84 (0.66-1.07) |
|  | L_den_, cat: <48 dB | 32 | [ref] | [ref] | [ref] | [ref] | [ref] | [ref] |
|  | L_den_, cat: 48-58 dB | 80 | 0.85 (0.59-1.23) | 0.78 (0.53-1.15) | [unavail.] | [unavail.] | 0.72 (0.48-1.09) | 0.72 (0.48-1.08) |
|  | L_den_, cat: >58 dB | 36 | 0.88 (0.57-1.35) | 0.79 (0.50-1.25) | [unavail.] | [unavail.] | 0.71 (0.39-1.27) | 0.68 (0.40-1.16) |

*Abbreviations: cat, categorical (tertiles); dB, decibel; L_den_, annual mean 24-hour road traffic noise levels; N, sample size; ref, categorical reference in model; unavail., PM_2.5_/PM_10_ data unavailable
Model estimates are hazard ratios and 95% confidence intervals [HR (95% CI)]
^*^ 10 dB increments of L_den_Crude model: adjusted for age (calendar year / underlying time) and year of cohort entry (inclusion year: 1993/1999);
Fully-adjusted model: Crude model + physical activity, marital status, alcohol, smoking, and fruit consumption.*

**Table S III –** Likelihood ratio tests of significance for non-linear exposures

| **Exposure variable** | **Exposure window** | **P-value** |
| --- | --- | --- |
| L_den_ | 1 | 0.45 |
| L_den_ | 3 | 0.17 |
| L_den_ | 23 | 0.28 |
| PM_2.5_ | 1 | 0.50 |
| PM_2.5_ | 3 | 0.47 |
| PM_2.5_ | 23 | [unavail.] |
| PM_10_ | 1 | 0.72 |
| PM_10_ | 3 | 0.99 |
| PM_10_ | 23 | [unavail.] |
| NO_2_ | 1 | 0.24 |
| NO_2_ | 3 | 0.31 |
| NO_2_ | 23 | 0.23 |
| NO_x_ | 1 | 0.21 |
| NO_x_ | 3 | 0.02 |
| NO_x_ | 23 | 0.22 |

*Abbreviations: L_den_, annual mean 24-hour road traffic noise levels; unavail., PM_2.5_/PM_10_ data unavailable
To check for linearity of exposures, we evaluated the shapes of the exposure-response functions by a likelihood ratio test of the significance of the non-linear part of the pollutant measure. Low p-values indicate statistically significant deviations from linearity.*

**Table S IV** - Associations between 1-, 3-, and 23-year mean road traffic noise level (53 dB cut-off) and incident Stroke (all, ischemic, hemorrhagic) among the Danish Nurse Cohort

| **Stroke sub-type** | **L_den_ window** | **Model type** | | | | | |
| --- | --- | --- | --- | --- | --- | --- | --- |
|  |  | **Crude** | **Fully-adjusted** | **Fully-adjusted + PM_2.5_** | **Fully-adjusted + PM_10_** | **Fully-adjusted + NO_2_** | **Fully-adjusted + NO_x_** |
| **All** | 1 | 1.07 (0.95-1.20) | 1.05 (0.93-1.18) | 0.95 (0.82-1.09) | 0.98 (0.86-1.13) | 0.88 (0.74-1.05) | 0.91 (0.77-1.07) |
|  | 3 | 1.08 (0.96-1.21) | 1.05 (0.93-1.19) | 0.96 (0.83-1.11) | 1.00 (0.87-1.16) | 0.92 (0.76-1.10) | 0.93 (0.79-1.10) |
|  | 23 | 1.10 (0.97-1.26) | 1.08 (0.93-1.24) | [unavail.] | [unavail.] | 0.97 (0.79-1.19) | 0.99 (0.82-1.19) |
| **Ischemic** | 1 | 1.09 (0.97-1.23) | 1.07 (0.94-1.22) | 0.96 (0.83-1.12) | 1.01 (0.87-1.17) | 0.89 (0.74-1.08) | 0.94 (0.79-1.12) |
|  | 3 | 1.11 (0.98-1.25) | 1.09 (0.95-1.24) | 1.00 (0.85-1.17) | 1.05 (0.90-1.22) | 0.95 (0.78-1.15) | 0.98 (0.82-1.17) |
|  | 23 | 1.13 (0.98-1.29) | 1.11 (0.96-1.29) | [unavail.] | [unavail.] | 1.00 (0.81-1.25) | 1.04 (0.85-1.27) |
| **Hemorrhagic** | 1 | 0.93 (0.66-1.29) | 0.89 (0.63-1.27) | 0.84 (0.56-1.25) | 0.83 (0.56-1.23) | 0.81 (0.49-1.35) | 0.72 (0.45-1.16) |
|  | 3 | 0.90 (0.64-1.26) | 0.85 (0.59-1.22) | 0.73 (0.48-1.11) | 0.74 (0.49-1.12) | 0.72 (0.43-1.22) | 0.67 (0.41-1.08) |
|  | 23 | 0.94 (0.64-1.39) | 0.86 (0.57-1.31) | [unavail.] | [unavail.] | 0.76 (0.42-1.37) | 0.68 (0.40-1.19) |

*Abbreviations: L_den_, annual mean 24-hour road traffic noise levels; unavail., PM_2.5_/PM_10_ data unavailable
Model estimates are hazard ratios and 95% confidence intervals [HR (95% CI)]
Crude model: adjusted for age (calendar year / underlying time) and year of cohort entry (inclusion year: 1993/1999);
Fully-adjusted model: Crude model + physical activity, marital status, alcohol, smoking, and fruit onsumption.*

**Table S V**- Associations between 1-, 3-, and 23-year mean road traffic noise level (58 dB cut-off) and incident Stroke (all, ischemic, hemorrhagic) among the Danish Nurse Cohort

| **Stroke sub-type** | **L_den_ window** | **Model type** | | | | | |
| --- | --- | --- | --- | --- | --- | --- | --- |
|  |  | **Crude** | **Fully-adjusted** | **Fully-adjusted + PM_2.5_** | **Fully-adjusted + PM_10_** | **Fully-adjusted + NO_2_** | **Fully-adjusted + NO_x_** |
| **All** | 1 | 1.13 (0.95-1.36) | 1.12 (0.92-1.36) | 0.95 (0.76-1.19) | 1.00 (0.80-1.25) | 0.87 (0.66-1.16) | 0.88 (0.66-1.16) |
|  | 3 | 1.14 (0.95-1.37) | 1.12 (0.92-1.36) | 0.95 (0.75-1.20) | 1.01 (0.80-1.28) | 0.89 (0.67-1.19) | 0.89 (0.67-1.18) |
|  | 23 | 1.09 (0.86-1.37) | 1.04 (0.81-1.34) | [unavail.] | [unavail.] | 0.78 (0.55-1.11) | 0.79 (0.55-1.12) |
| **Ischemic** | 1 | 1.18 (0.98-1.43) | 1.17 (0.96-1.44) | 0.99 (0.78-1.26) | 1.06 (0.83-1.34) | 0.91 (0.67-1.23) | 0.96 (0.71-1.29) |
|  | 3 | 1.19 (0.98-1.44) | 1.18 (0.96-1.45) | 1.02 (0.80-1.31) | 1.10 (0.86-1.41) | 0.95 (0.70-1.30) | 0.97 (0.72-1.32) |
|  | 23 | 1.12 (0.88-1.44) | 1.10 (0.84-1.43) | [unavail.] | [unavail.] | 0.82 (0.56-1.19) | 0.86 (0.59-1.24) |
| **Hemorrhagic** | 1 | 0.81 (0.46-1.44) | 0.76 (0.42-1.39) | 0.69 (0.35-1.35) | 0.66 (0.34-1.29) | 0.62 (0.27-1.45) | 0.47 (0.20-1.10) |
|  | 3 | 0.77 (0.43-1.41) | 0.72 (0.38-1.34) | 0.57 (0.27-1.17) | 0.55 (0.27-1.14) | 0.55 (0.23-1.33) | 0.43 (0.18-1.05) |
|  | 23 | 0.83 (0.40-1.72) | 0.72 (0.33-1.56) | [unavail.] | [unavail.] | 0.58 (0.20-1.64) | 0.44 (0.15-1.27) |

*Abbreviations: L_den_, annual mean 24-hour road traffic noise levels; unavail., PM_2.5_/PM_10_ data unavailable
Model estimates are hazard ratios and 95% confidence intervals [HR (95% CI)]
Crude model: adjusted for age (calendar year / underlying time) and year of cohort entry (inclusion year: 1993/1999);
Fully-adjusted model: Crude model + physical activity, marital status, alcohol, smoking, and fruit consumption.*

**Table S VI** - Effect modification of the association between L_den_ (continuous, 1-year mean, per 10 dB increase) and incidence of ischemic stroke in the Danish Nurse Cohort

| **Variable** | **Level** | **Hazard Ratio** | **p-value** |
| --- | --- | --- | --- |
| **PM_2.5_** | <19.5 | 1.03 (0.94-1.12) | 0.8849458 |
|  | 19.5-23.2 | 1.08 (0.81-1.45) |  |
|  | >23.2 | 1.14 (0.66-1.98) |  |
| **NO_2_** | <19.5 | 1.01 (0.93-1.11) | 0.9304859 |
|  | 19.5-23.2 | 1.13 (0.50-2.53) |  |
|  | >23.2 | 1.10 (0.64-1.89) |  |
| **Obesity** | Not obese | 1.06 (0.98-1.15) | 0.4841997 |
|  | Obese | 0.96 (0.73-1.26) |  |
| **Shiftwork type** | Day / Evening / Rotating | 1.03 (0.92-1.15) | 0.4780752 |
|  | Night | 0.91 (0.66-1.25) |  |
| **Hypertension** | No | 1.05 (0.96-1.15) | 0.9139204 |
|  | Yes | 1.04 (0.88-1.23) |  |
| **Acute myocardial infarction** | No | 1.05 (0.97-1.13) | 0.8788412 |
|  | Yes | 1.00 (0.58-1.73) |  |
| **Diabetes mellitus** | No | 1.07 (0.99-1.16) | 0.0137913 |
|  | Yes | 0.62 (0.41-0.93) |  |
| **Leisure-time  physical activity** | Low | 1.19 (0.92-1.55) | 0.3926699 |
|  | Medium | 1.07 (0.97-1.18) |  |
|  | High | 0.98 (0.84-1.14) |  |
| **Urbanicity degree** | Urban | 1.05 (0.89-1.23) | 0.3114485 |
|  | Suburban | 0.92 (0.77-1.10) |  |
|  | Rural | 1.08 (0.96-1.21) |  |
| **Hormone replacement therapy use** | Never | 1.04 (0.94-1.14) | 0.9114536 |
|  | Past | 1.07 (0.87-1.31) |  |
|  | Current | 1.08 (0.91-1.28) |  |
| **Occupational status** | Working | 1.01 (0.91-1.13) | 0.0036382 |
|  | Homeworker | 1.07 (0.59-1.94) |  |
|  | Retired | 1.08 (0.96-1.21) |  |
|  | Unemployed | 67.84 (3.06-1505.21) |  |
|  | Other | 3.03 (0.53-17.26) |  |

Table S VII - Overview of studies on associations between long-term exposure to road traffic noise and stroke

| Authors (year) | Study/Area/Samplesize (N) | Stroke Defintion | Noise exposure window | Noise exposure level^+^ | Air pollution (potential confounder) | Effect Estimates in Total Population | Effect Estimates in Males | Effect Estimates in  Females |
| --- | --- | --- | --- | --- | --- | --- | --- | --- |
| (Cole-Hunter et al. 202X [current study]) | Danish Nurse Cohort (DNC),  Denmark  N=23,423 | ICD-8: 431, 432, 433, 434, 436;  ICD-10: I61, I63, I64 | 1-, 3-, 23-year | 52.7 dB  [SD: 8.2 dB] | PM_2.5_, PM_10_, NO_2_, NO_x_ | N/A | N/A | HR (95% CI): 1-year  *Overall*  1.06 (0.99, 1.13)  *Ischemic*  1.06 (0.99, 1.14)  *Hemorrhagic*  1.04 (0.86, 1.26) |
| (Andersson *et al.*, 2020) | Swedish Primary Prevention Study cohort, Sweden  1970–1973/2011  N=6304 | ICD-8: 431–436;  ICD-10: I61–64 | 1-year | 55 dB | NO_x_ | N/A | HR (95% CI): 1-year  1.07 (0.85–1.36) | N/A |
| (Thacher *et al.*, 2020) | Danish Diet, Cancer, and Health cohort,  Denmark  1993-2015 | ICD-10: I60–I64 | 1-, 5-, 10-year | [Median: 55.8]  44.3–69.3 dB | PM_2.5_, NO_2_ | HR (95% CI): 1-; 5-; 10-year  1.16 (1.04, 1.29);  1.18 (1.05, 1.32);  1.19 (1.06, 1.33) | N/A | N/A |
| (Klompmaker *et al.*, 2019) | Dutch Public Health Monitor 2012 (PHM),  Netherlands  2002-2012  N=354,827 | Self-reported physician-diagnosed stroke | Daily (cross-sectional) | [Median (IQR): 53.3 (7.5)] | PM, NO_2_ | OR (95% CI)  1.01 (0.98, 1.03) | N/A | N/A |
| (Pyko *et al.*, 2019) | Stockholm County,* Sweden  1987-2011  N=20,012 | ICD-8: 430–436; ICD-10: I60–I65 [including ischaemic  (ICD-8: 433X–435X;  ICD-10: I63X, I65X, I66X)] | 5-year | 46.8 dB  30–75 dB | BC, PM_2.5_ | HR (95% CI): 5-year  1.00 (0.92, 1.09) | HR (95% CI): 5-year  0.99 (0.89 to 1.11) | HR (95% CI): 5-year  1.02 (0.91 to 1.14) |
| (Vivanco-Hidalgo *et al.*, 2019) | BASICMAR prospective register, Barcelona, Spain N=2761 | Initial (acute ischemic) stroke severity (National Institute of Health Stroke Scale [NIHSS]: 0–42) | 1-year / Annual | 65 dB | PM_2.5_ (+ residential surrounding greenness [NDVI]) | OR (95% CI) (NDVI 100m):  *Dichotomous NIHSS >5*: 1.15 (1.02-1.30)  *Continuous NIHSS*: 0.05 (0.00,0.10) | N/A | N/A |
| (Cai *et al.*, 2018) | BioSHaRE-EU project,  UK  N= 355,732 | Hemorrhagic stroke (ICD-8: 431; ICD-10: I60, I61, I62);  Ischemic (ICD- 8:433,434; ICD-10:I63) | 1-year | 55.2 dB  48.4–65.8 dB | PM, NO_2_ | Hemorrhagic  *Crude*  0.938 (0.845, 1.041)  *Adjusted*  0.934 (0.841, 1.036)  Ischemic  *Crude*  0.999 (0.943, 1.057)  *Adjusted*  0.996 (0.941, 1.055) | N/A | N/A |
| (Seidle*r et al*>, 2018) | Frankfurt airport vicinity insured by one of three large statutory health insurance funds between 2005 and 2010  N= 1,026,670 | ICD-10: Intracerebral hemorrhage (I61); cerebral infarction (I63); stroke, not specified as hemorrhage or infarction (I64) | 24-hour average (L _pAeq,24h_) and maximum night-time (L _pAeq,night_) sound level | Linear (per 10 dB) and categorical (40-<45, 45-<50, 50-<55, 55-<60, 60-<65, 65-<70, >70) dB | N/A | Per 10 dB  OR (95% CI): 1.017 (1.003, 1.032)  Categorical (dB)  <55: ~1.00  55-<60: 1.05 (1.00, 1.11)  60-<65: 1.01 (0.95, 1.07)  65-<70: 1.07 (1.00, 1.14)  ≥70: 1.02 (0.91, 1.14) | N/A | N/A |
| (Héritier *et al.*, 2017) | Swiss National Cohort (SNC)  N= 4,415,206 | ICD-10: I60–I64  [Hemorrhagic (ICD-10: I60–I62);  Ischemic  (ICD-10: I63)] | 1-year | ? dB | NO_2_ | HR (95% CI): 1-year  *Crude*  1.005 (0.989, 1.022)  *Adjusted*  1.011 (0.993, 1.028) | N/A | N/A |
| (Dzhambov and Dimitrova, 2016) | Meta-analysis: Several | Several |  |  |  | RR (95% CI):  1.03 (0.87, 1.22) | N/A | N/A |
| (Halonen *et al.*, 2015) | Greater London,  UK  N=8,610,000  [Adults, >25 years;  Elderly, > 75 years] | ICD-10: I61, I63, I64 | 1-year  L_Aeq,16h_  L_night_  <55 dB / 55-60 dB / >60 dB | L_Aeq,16h_  57.7 dB  (54.9-64.6 dB)^§^  L_night_  52.4 dB  (49.3-59.7 dB)^§^ | PM_2.5_, NO_x_ | RR (95% CI):  L_Aeq,16h_  *>60 dB, Adults*  1.05 (1.02, 1.09)  L_night_  *>60 dB, Adults*  1.01 (0.98, 1.05) | N/A | N/A |
| (Sørensen *et al.*, 2014) | Danish Diet, Cancer, and Health cohort,  Denmark  1993-2010  N=51,569 | ICD-10: I60, I61, I63, I64 | 1-, 5-, 10-year | [Median: 56.5 dB]  (48.9-70.1 dB) | NO_x_, NO_2_ | IRR (95% CI): 1-, 5-, 10-year  *Ischemic*  1: 1.16 (1.07, 1.24);  5: 1.14 (1.06, 1.23);  10: 1.12 (1.03, 1.21); | N/A | N/A |
| (Sørensen *et al.*, 2011) | Danish Diet, Cancer, and Health cohort,  Denmark  1993-2006  N=51,485 | ICD-8: 431.0, 431.9, 432.0, 432.9, 433.09, 433.99, 434.09, 434.99, 436.0,  436.9  ICD-10: DI61, DI63 | 1-year | ? | NO_x_ | IRR (95% CI):  *Crude*  1.18 (1.11, 1.26)  *Adjusted*  1.14 (1.03, 1.25) | IRR (95% CI):  *Crude*  1.20 (1.10, 1.30)  *Adjusted*  1.14 (1.02, 1.27) | IRR (95% CI):  *Crude*  1.17 (1.05, 1.29)  *Adjusted*  1.13 (1.00, 1.28) |

**Supplemental references** (from Table S VII)

Andersson, E. M. *et al.* (2020) ‘Road traffic noise, air pollution and cardiovascular events in a Swedish cohort’, *Environmental Research*, 185(March), p. 109446. doi: 10.1016/j.envres.2020.109446.

Cai, Y. *et al.* (2018) ‘Road traffic noise, air pollution and incident cardiovascular disease: A joint analysis of the HUNT, EPIC-Oxford and UK Biobank cohorts’, *Environment International*, 114(February), pp. 191–201. doi: 10.1016/j.envint.2018.02.048.

Dzhambov, A. M. and Dimitrova, D. D. (2016) ‘Exposure-response relationship between traffic noise and the risk of stroke: A systematic review with meta-analysis’, *Arhiv za Higijenu Rada i Toksikologiju*, 67(2), pp. 136–151. doi: 10.1515/aiht-2016-67-2751.

Halonen, J. I. *et al.* (2015) ‘Road traffic noise is associated with increased cardiovascular morbidity and mortality and all-cause mortality in London’, *European Heart Journal*, 36(39), pp. 2653–2661. doi: 10.1093/eurheartj/ehv216.

Héritier, H. *et al.* (2017) ‘Transportation noise exposure and cardiovascular mortality: a nationwide cohort study from Switzerland’, *European Journal of Epidemiology*, 32(4), pp. 307–315. doi: 10.1007/s10654-017-0234-2.

Klompmaker, J. O. *et al.* (2019) ‘Associations of combined exposures to surrounding green, air pollution, and road traffic noise with cardiometabolic diseases’, *Environmental Health Perspectives*, 127(8), pp. 1–15. doi: 10.1289/EHP3857.

Pyko, A. *et al.* (2019) ‘Long-term transportation noise exposure and incidence of ischaemic heart disease and stroke: A cohort study’, *Occupational and Environmental Medicine*, 76(4), pp. 201–207. doi: 10.1136/oemed-2018-105333.

Seidler, A. L. *et al.* (2018) ‘The effect of aircraft, road, and railway traffic noise on stroke - Results of a case-control study based on secondary data’, *Noise and Health*, 20(95), pp. 152–161. doi: 10.4103/nah.NAH_7_18.

Sørensen, M. *et al.* (2011) ‘Road traffic noise and stroke: A prospective cohort study’, *European Heart Journal*, 32(6), pp. 737–744. doi: 10.1093/eurheartj/ehq466.

Sørensen, M. *et al.* (2014) ‘Combined effects of road traffic noise and ambient air pollution in relation to risk for stroke?’, *Environmental Research*, 133, pp. 49–55. doi: 10.1016/j.envres.2014.05.011.

Thacher, J. D. *et al.* (2020) ‘Long-term residential road traffic noise and mortality in a Danish cohort’, *Environmental Research*, 187(January), p. 109633. doi: 10.1016/j.envres.2020.109633.

Vivanco-Hidalgo, R. M. *et al.* (2019) ‘Association of residential air pollution, noise, and greenspace with initial ischemic stroke severity.’, *Environmental Research*, 179(August), p. 108725. doi: 10.1016/j.envres.2019.108725.
